# Protein Conservation and Variation Suggest Mechanisms of Cell Type-Specific Modulation of Signaling Pathways

**DOI:** 10.1371/journal.pcbi.1003659

**Published:** 2014-06-12

**Authors:** Martin H. Schaefer, Jae-Seong Yang, Luis Serrano, Christina Kiel

**Affiliations:** 1 EMBL/CRG Systems Biology Research Unit, Centre for Genomic Regulation (CRG), Barcelona, Spain; 2 Universitat Pompeu Fabra (UPF), Barcelona, Spain; 3 Institució Catalana de Recerca i Estudis Avançats (ICREA), Barcelona, Spain; Northeastern University, United States of America

## Abstract

Many proteins and signaling pathways are present in most cell types and tissues and yet perform specialized functions. To elucidate mechanisms by which these ubiquitous pathways are modulated, we overlaid information about cross-cell line protein abundance and variability, and evolutionary conservation onto functional pathway components and topological layers in the pathway hierarchy. We found that the input (receptors) and the output (transcription factors) layers evolve more rapidly than proteins in the intermediary transmission layer. In contrast, protein expression variability decreases from the input to the output layer. We observed that the differences in protein variability between the input and transmission layer can be attributed to both the network position and the tendency of variable proteins to physically interact with constitutively expressed proteins. Differences in protein expression variability and conservation are also accompanied by the tendency of conserved and constitutively expressed proteins to acquire somatic mutations, while germline mutations tend to occur in cell type-specific proteins. Thus, conserved core proteins in the transmission layer could perform a fundamental role in most cell types and are therefore less tolerant to germline mutations. In summary, we propose that the core signal transmission machinery is largely modulated by a variable input layer through physical protein interactions. We hypothesize that the bow-tie organization of cellular signaling on the level of protein abundance variability contributes to the specificity of the signal response in different cell types.

## Author Summary

Cell function is determined by highly organized networks of biological molecules. An important class of protein pathways maintains the transmission of signals from the cell membrane to the nucleus. These signaling pathways are reused for different purposes at an evolutionary scale and in different cell types of the same organism. However, it is largely unknown how this flexibility is achieved and how this flexibility is balanced with the high degree of evolutionary conservation of some signaling proteins and the need for robustness against intra- and extra-cellular perturbations.We show how functional roles of signaling proteins determine patterns of evolutionary conservation, protein abundance (the average over different human cell lines and its variability) and disease mutations. Projecting pathway annotations on protein-protein interaction (PPI) networks, a picture emerges in which PPIs between variable and less conserved receptors and stable and conserved proteins of the core signal transmission machinery largely modulate signaling activity in a tissue-specific manner. This has important implications for the distribution of disease mutations in signaling pathways, which need to be considered for the understanding of their effect.

## Introduction

Proteins do not act in isolation but interact with other proteins to fulfill important cellular functions. Often proteins are organized into pathways, which are tightly controlled cascades of protein binding events (and those of other biomolecules). One important cellular function controlled by pathways is the transmission of extra-cellular signals from the cell membrane to the nucleus to provoke a response to changes in the environment of the cell. Signaling pathways are often active in many different cell types and are conserved at a large evolutionary scale [1]. Therefore, the characterization of mechanisms by which these ubiquitous pathways achieve specificity and fulfill largely different functions in different cell types or organisms is of crucial importance.

One characteristic of signaling pathways is the bow-tie (or hourglass) architecture in which signals sensed by receptors converge onto a core consisting of a smaller number of proteins followed by a diverse response of transcription factors. The bow-tie property has been observed in different human signaling pathways such as those downstream of epidermal growth factor receptor [2] and of toll-like receptor [3]. It is generally believed to confer pathways with robustness and evolvability by buffering input signals and modularizing the response [4]. However, robustness by hierarchy comes to a price: mutations in the central core proteins might easily hijack the behavior of the entire system [5].

The robustness of pathways needs to be in balance with flexibility allowing pathways to vary their response to similar stimuli at different time points or in different cell types of the same organism. One intuitive though largely unexplored link to the bow tie model comes from investigations of protein-protein interaction (PPI) networks associated with gene expression information: it was observed that tissue-specific proteins tend to bind to core cellular proteins, possibly to modulate housekeeping cellular processes in a cell type-specific manner [6].

The mechanistic understanding about how activation of the same signaling pathways can lead to cell type-specific responses is rather anecdotal and involves diverse mechanisms such as cell type-specific feedback loops [7], different abundances of transcriptional cofactors [8], [9] or cell type-specific chromatin states [10]. However, it is largely unknown if there are functional or (network) topological signal protein classes that preferentially act as tissue-specific modulators of signaling. Therefore, we will explore here if we can adapt the bow-tie model to identify protein classes that show distinct evolutionary and abundance variability patterns.

Recently a mass spectroscopy analysis accomplished by Mann and co-workers [11] has determined absolute protein copy numbers for 11 common human cancer cell lines with high coverage. The analyzed cell lines covered distinct tissue origins, such as lung carcinoma, hepatoma, osteosarcoma, colon carcinoma, and leukemia [11]. Multiple technical replicates allow to make robust estimates of protein expression variability by contrasting the inter-cell line with the intra-cell line variability and, therefore, make this dataset a perfect choice for quantifying differences in protein expression among different cell types. Using this dataset and defining a measure of protein conservation covering a broad set of species, we systematically investigate patterns of protein expression variability and phylogenetic conservation in human pathways. We observe large differences in protein expression and in phylogenetic conservation between and within different human pathways. Focusing on human signaling, we identify components of signaling pathways with distinct properties in respect to these features. By incorporating germline and somatic disease mutations, we show how the thereby identified pathway components underlie different selective constraints.

## Results

### Protein abundance variability across cancer cell lines and healthy tissues

To estimate mean abundance and abundance variability of human proteins, we processed a recent proteomics study quantifying protein expression levels in 11 human cell lines [11]. Due to technical limitations of the mass spectrometry approach, lowly abundant proteins are associated with higher standard deviations (Figure S1A). To correct for this, we computed *F* values to estimate the biological variability among cell lines. *F* values were computed by dividing the between-cell variation by the within-cell variation. Thereby we successfully eliminated any dependencies between the protein abundance and variability caused by technical biases (see Figure S1B).

In this study, we used the *F* values computed on cancer cell lines to distinguish proteins that are stably expressed across different cell types from those showing more diverse abundance profiles. We validated the underlying assumption that we can generalize our observations made on cancer cell line data to healthy tissues by contrasting the computed *F* values with RNA (16 human tissues) [12] and protein (28 mouse tissues) measurements [13] (see Methods). In both cases there is generally a good agreement between protein variability across the cell lines and healthy tissues (Figure S2A–B). This supports the idea that we can generalize from cancer cell lines to healthy human tissues with respect to protein abundance variability.

### Relation between protein abundance and phylogenetic conservation

**Figure 1:**
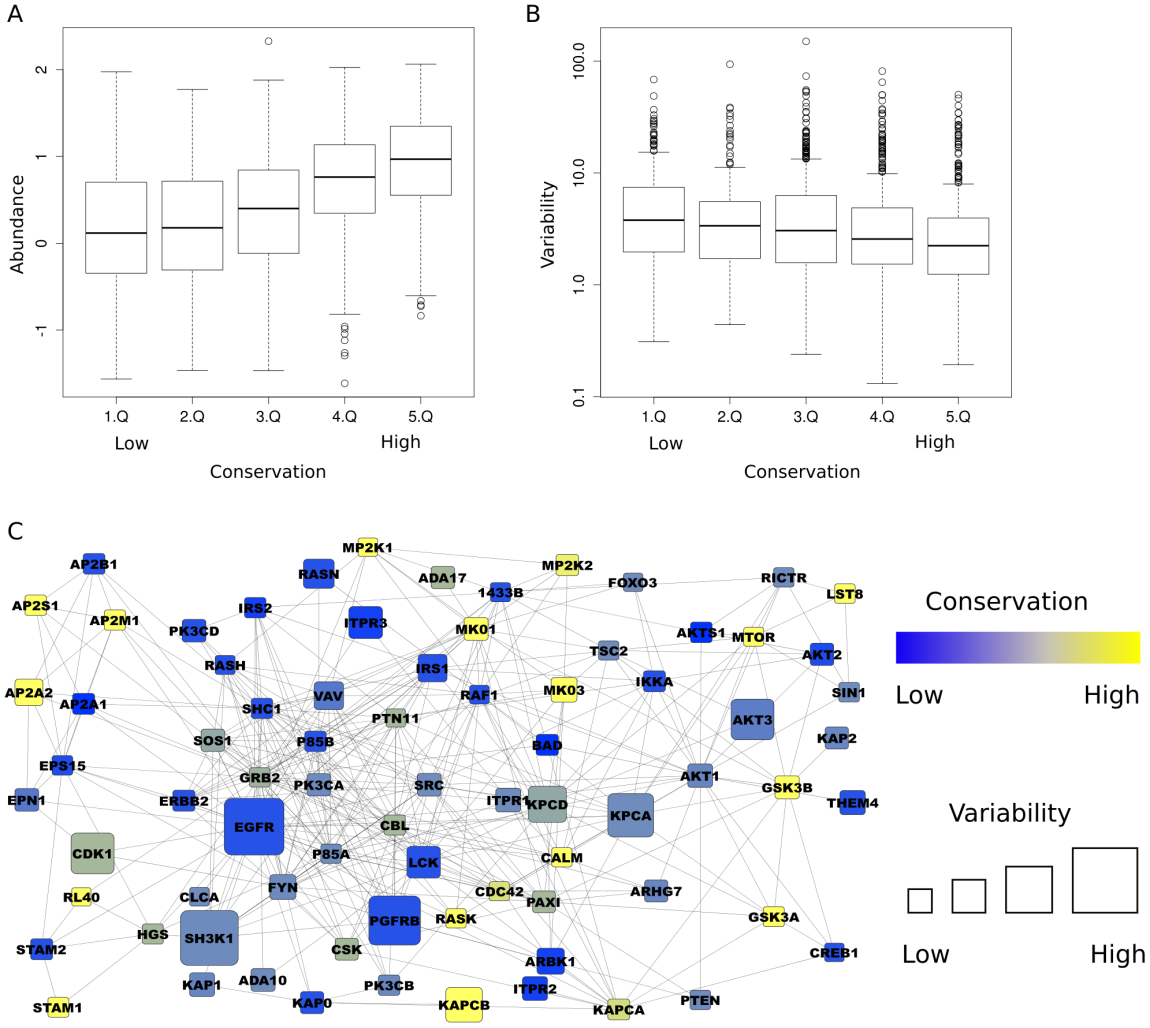
Relation between protein conservation, abundance and variability. (**A**) Conservation is correlated with protein abundance (correlation = 0.42, *P*<0.0001) and (**B**) inversely related to protein abundance variability (correlation = −0.11, *P*<0.0001). The conservation values have been binned into 20% quantiles (Q). (**C**) Human signaling pathways are composed of conserved proteins with low abundance variability and variable proteins with low conservation. The figure shows the largest cluster of interacting proteins from the human EGFR pathway (participating proteins as indicated by Reactome, PPIs taken from HIPPIE). The node size is proportional to the abundance variability and conservation is encoded by a color gradient ranging from blue (non-conserved) to yellow (conserved).

To analyze how protein conservation relates to protein expression abundance and diversity of proteins involved in human pathways, we analyzed the conservation of all proteins in the expert-curated Reactome database [14] in selected species from Plants, Yeasts, Worms, Insects, Fishes, Birds, and Mammals. We transformed the information in which species a human protein is conserved, as indicated by HomoloGene [15], into a phylogenetic tree-based conservation score (see Methods and Figure S3), which increases linearly with the amount of species in which homologous proteins are found and estimates of evolutionary distance separating these species from each other. We observed significant positive correlation between the evolutionary conservation of human proteins and their mean abundance (see Figure 1A) or negative correlation in their variability in the different cell lines analyzed (see Figure 1B and an example on the EGFR/MAPK pathway containing both variable/lowly conserved and stably expressed/conserved proteins in Figure 1C).

### Protein abundance, variation, and conservation of cellular processes

To identify cellular processes that differ in their phylogentic conservation and cross-cell variability, we selected all Reactome pathways that are expected to be general and not restricted to only some cell types (ten pathways): Cell cycle, DNA replication, and chromosome maintenance [DR], Extracellular matrix organization [MO], Gene expression and RNA processing [GE], Membrane trafficking [MT], Metabolism [MB], Signal transduction [ST], Apoptosis [AP], Developmental Biology [DB], Transmembrane transport of small molecules [TM], and Cell-Cell communication [CO]. Several other Reactome pathways are restricted to very specific body cell types (e.g., Neuronal system and Muscle contraction), or are of low coverage (e.g, Circadian clock proteins), and were neglected for further analysis (for details on the selection see Materials and Methods and Table S1). We associated all members of the ten pathways with mean protein abundance, abundance variability and phylogenetic conservation values.

A fraction of the proteins (18.7% from the 4069 Reactome proteins that we could associate with at least one of the investigated features) participates in more than one pathway. We compared the distributions of mean abundance, abundance variability and phylogenetic conservation among exclusive proteins associated only with one pathway to the distributions associated with proteins involved in several pathways. We observed that exclusive proteins are significantly less conserved (*P*<e^−16^; Wilcoxon-Mann-Whitney), more variable (*P*<e^−12^; Wilcoxon-Mann-Whitney) and less abundant (*P*<e^−8^; Wilcoxon-Mann-Whitney) (see Figure S4A–C).

**Figure 2:**
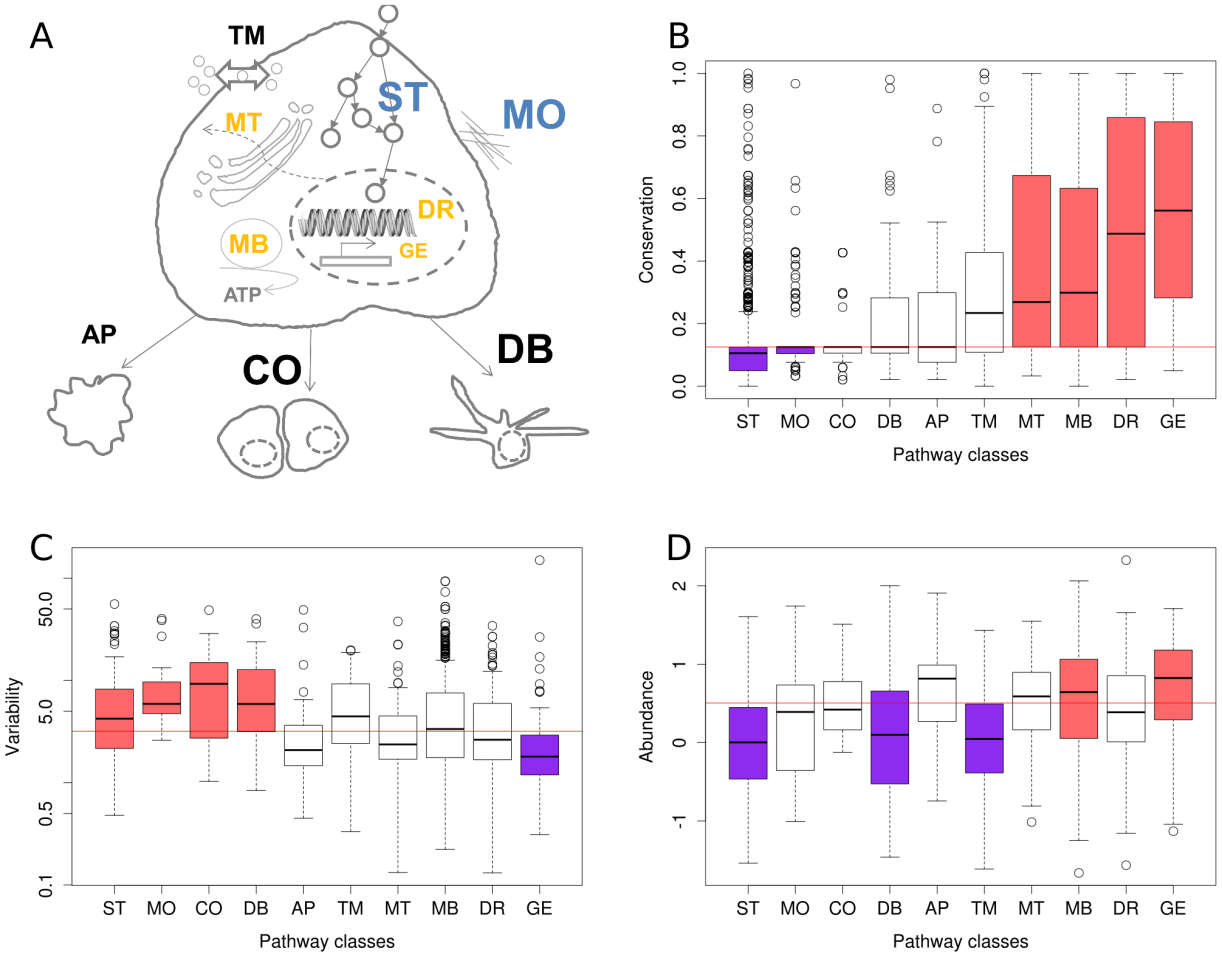
Protein abundance, variability and conservation associated with general cellular processes. We investigated properties of proteins involved in ten Reactome top-level pathways (**A**): Cell cycle, DNA replication, and chromosome maintenance [DR], Extracellular matrix organization [MO], Gene expression and RNA processing [GE], Membrane trafficking [MT], Metabolism [MB], Signal transduction [ST], Apoptosis [AP], Developmental Biology [DB], Transmembrane transport of small molecules [TM], and Cell-Cell communication [CO]. Several pathways were significantly higher conserved (yellow letters) or showed a significant lower conservation (blue letters). Similarly, several pathways showed significantly higher protein abundance variability (large font size) or significantly lower variability (small font size). (**B**–**D**) shows the precise distributions for the different investigated protein features. Colored boxes indicate a significantly (p<0.01; Mann-Whitney-Wilcoxon test) lower (purple) or higher (red) abundance/variability/conservation of the proteins within the class as compared to proteins not in the class. The horizontal red line marks the median value for all proteins in Reactome.

Next, to elucidate the common and variable elements in 11 cell types with respect to the ten Reactome pathways, we considered only proteins found exclusively in one pathway. We observed large differences with respect to the three investigated features among human functional pathway classes (Figure 2A). In general, we found two opposing groups of behavior. Housekeeping pathways (GE, MB, MT and DR) are enriched in conserved proteins (Figure 2B), have low to average variability (with GE being the only pathway class with a significant depletion in variability; Figure 2C) and (except for DR) higher abundance (GE and MB are significantly enriched in highly abundant proteins; Figure 2D). Specific pathways (ST, MO, CO, DB) tend to have less conserved proteins (ST and MO show a significant depletion; Figure 2B), have higher variability (Figure 2C) and less abundance (ST and DB are significantly associated with lower abundance; Figure 2D). The remaining two pathways (AP and TM) showed a rather average behavior, with exception of the significantly lower abundance of TM proteins. Signal transduction (ST) shows in all three categories (mean abundance, abundance variability and conservation) a significant deviation from random expectation and has a larger than average spread of the distribution of variability values (fourth highest inter-quartile range among the ten variability distributions). This indicates that while average variability of signaling proteins is high, we will also find a large proportion of proteins with low variability in signaling pathways. Hence, we studied the variable and constant parts of signaling pathways.

### Protein abundance, variation, and conservation in signaling-related pathways

To elucidate in more depth the protein abundance variability in signal transduction pathways, we investigated whether we can relate the molecular function of proteins in signaling pathways to their abundance, variability and conservation. For this purpose, we chose two complementary protein classification strategies and signaling resources. (a) We assigned, where non-ambiguously possible, signaling proteins in Reactome to one of the following Gene Ontology and UniprotKB categories: membrane-bound receptor, phosphatase, kinase, transcription factor, adaptors, and GTPase binding (see Methods for details). (b) We retrieved the full set of proteins and their classification into signaling-related sections (ligand, receptor, mediator, cofactor, transcription factor) from the signaling pathway database SignaLink [16], which is another manually curated resource classifying proteins into pathway sections based on their role in signal transmission and topological properties. For example, SignaLink distinguishes between mediators and cofactors of signal transduction, where mediators are core pathway members and the cofactors merely modulate the function of signaling proteins. We only considered signaling proteins in SignaLink that are uniquely assigned to one class. Due to different curation strategies (discussed in [16]), the sets of proteins associated with pathways largely differ between Reactome and SignaLink: we could automatically assign pathway functions to 802 proteins from Reactome while the SignaLink database contains 667 proteins with unique roles in signaling. The overlap between the two sets consists of only 80 proteins. The differences in protein composition and in the way proteins are associated with pathway functions allow us to study the evolution and expression of signaling proteins on two largely independent datasets.

**Figure 3:**
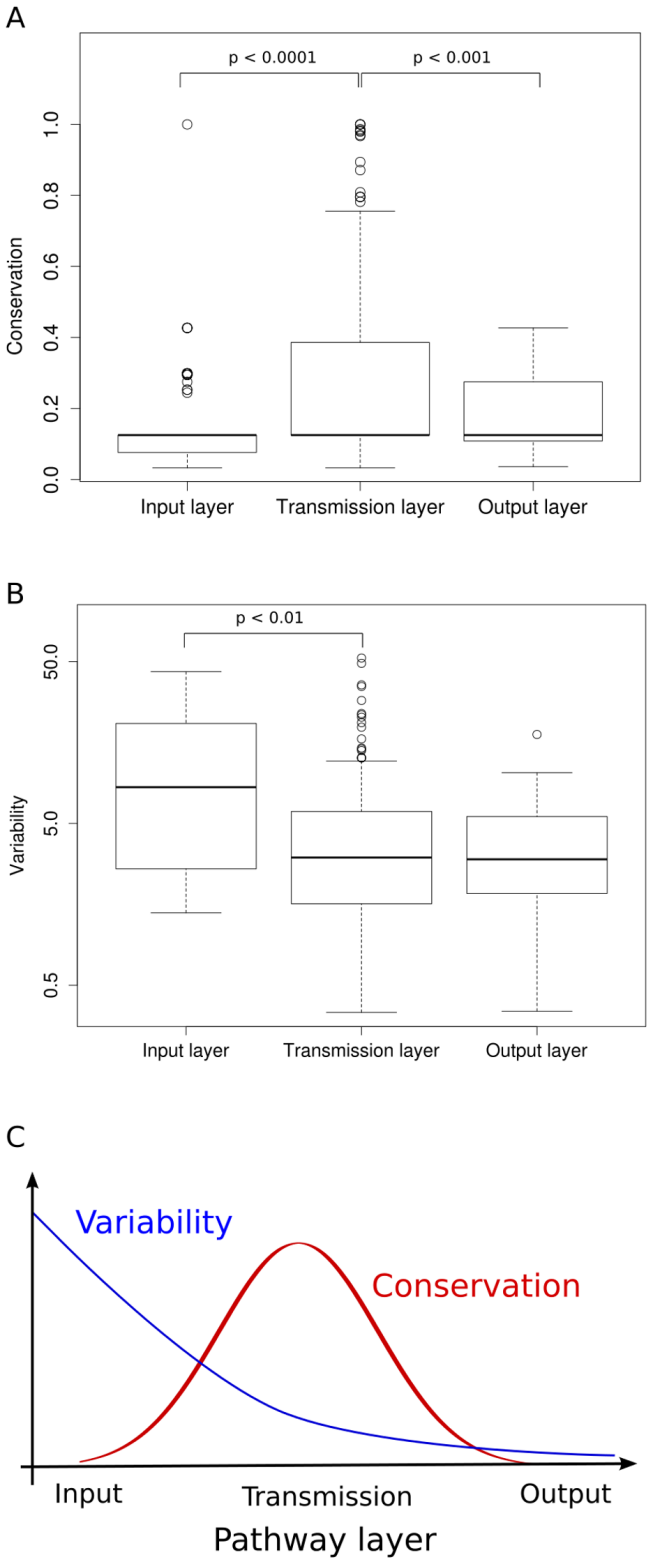
Conservation and abundance variability of components of human signaling pathways. Different components of signaling pathways show significant differences with respect to conservation (**A**) and protein abundance variability (**B**). Layers are generated by pooling different functional pathway classes. Here, we show the values for signaling proteins and their annotations from the Signalink database. We assigned receptors to the input layer, transcription factors to the output and mediators and cofactors (that are neither receptors nor transcription factors) to the transmission layer. Trends are schematized in (**C**).

We observed large differences in conservation, mean abundance and abundance variation for different classes within both sets of annotated signaling proteins (see Figure S5). Next, we pooled all functional and topological classes into three layers: input (receptors), transmission (SignaLink: mediators and cofactors; Reactome: kinases, phosphatases, adaptors and GTPase binding proteins) and output (transcription factors). We compared the resulting feature distributions. With respect to protein abundance variability we observed for both the Reactome and the SignaLink proteins significantly higher values associated with the input layer than with the transmission layer (*P*<0.01, Wilcoxon-Mann-Whitney). The difference between the transmission and the output layer was for both data sources not (Reactome) or only marginally (*P* = 0.03; Wilcoxon-Mann-Whitney; SignaLink) significant. The conservation of proteins of the transmission layer was significantly larger than of proteins of both the input and the output layer (for all comparisons: *P*<0.00001; Wilcoxon-Mann-Whitney). In summary, taking a mechanistic view on signaling pathways where an input layer receives signals from the environment, a transmission layer integrates and proceeds the signal and an output layer orchestrates the transcriptional response, two patterns emerge: (i) In terms of conservation, we see a bell shaped curve with a high conservation of the transmission layer and lower conservation of the input and output layer (Figure 3A and S5). (ii) With respect to protein abundance variability, signaling pathways show a gradient with decreasing variability from the input to the output layer (Figure 3B). There is a sharp drop in variability between the input and the transmission layer while the transmission and the output layer are rather similar in terms of variability (Figure 3B). These results are schematized in Figure 3C. The difference in terms of protein abundance between different functional classes was less pronounced. Interestingly, the variability and conservation of mediators and cofactors of signaling is almost the same (Wilcoxon-Mann-Whitney test does not yield *P*<0.05). This suggests that modulators in the transmission layer contribute less to cell type specific differences.

We also compared the investigated features associated with proteins exclusively members of one signaling pathway (1253 proteins) to those associated with proteins re-used in several signaling pathways (235 proteins) (see Figure S4D–F). We observed a significantly higher conservation of proteins that are members of several signaling pathways (*P*<e^−16^; Wilcoxon-Mann-Whitney), while mean abundance and abundance variability did not show significant differences between the protein sets. This is in agreement with the higher number of proteins of the transmission layer among the proteins associated with multiple pathways (e.g., 14 adaptors and 49 kinases, which exceeds random expectation 3- and 1.5-fold, respectively).

### Protein interactions between the input and the transmission layer

To investigate how our observation of a lowly variable and strongly conserved transmission layer might help to understand general principles by which signaling pathways are modulated in a tissue-specific manner, we overlaid our sets of signaling proteins (merged from Reactome and SignaLink) with PPI network data from the database HIPPIE [17], [18]. As we observed the highest protein abundance variability in the input layer (Figure 3B), we hypothesized that this variability affects the dynamics of physical interactions between the input and the transmission layer (by removing signaling links in certain tissues or modulating competition for binding in others).

**Figure 4:**
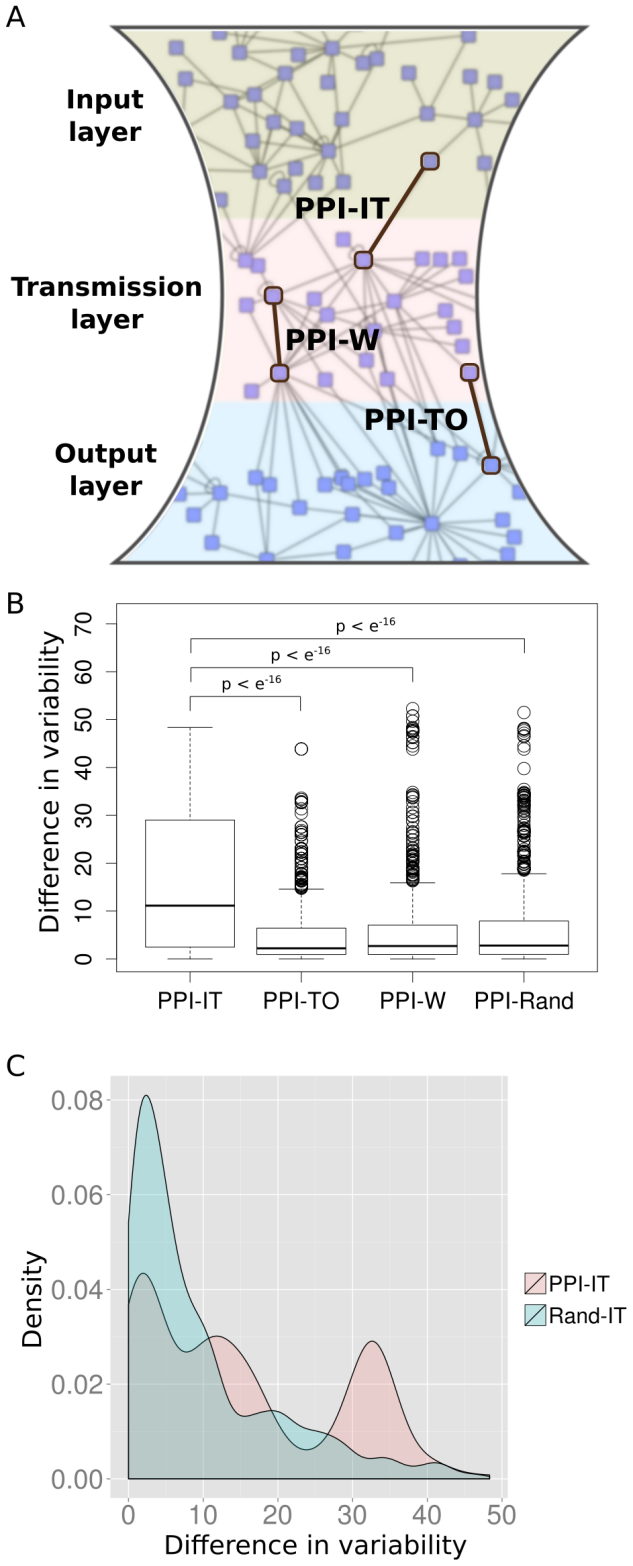
Absolute variability difference between interacting proteins between or within signaling pathway layers. We considered PPIs where one protein is from the input layer and the other one from the transmission layer (PPI-IT), interacting proteins where one is from the transmission layer and the other one from the output layer (PPI-TO), interacting proteins where both proteins are from the same layer (PPI-W) and random PPIs (PPI-Rand) (**A**). The distribution of differences between the input and the transmission layer contains significantly larger values than all other distributions (**B**). To exclude that this observation is only caused by the higher variability among proteins in the input layer as compared to proteins in the transmission layer, we compared the distribution of difference in variability associated with PPI-IT to randomly sampled, non-interacting protein pairs where one protein is in the input layer and the other one is in the transmission layer (Rand-IT) (**C**). Again, the PPI-IT distribution is significantly larger.

We tested the hypothesis that PPIs between input and transmission layer tend to happen between proteins with a larger difference in variability than for PPIs between the transmission and the output layer, within one layer, or randomly chosen PPIs (Figure 4A). To test this, we randomly sampled interacting protein pairs between and within the specified layer (each distribution of differences in protein abundance variability consisted of 1000 randomly sampled interacting protein pairs). We found the largest difference between the variability of the interacting proteins for PPIs between the input and the transmission layer (Figure 4B). The distribution of differences in variability was significantly larger than all other distributions (*P*<10^−16^; Wilcoxon-Mann-Whitney). As the difference in variability is highest between the input and the transmission layer, the results met our expectation.

To test if the observed difference in variability between interacting proteins between the input and the transmission layer can be solely attributed to the membership of the participating proteins in different layers, we compared the distribution of variability differences for interacting proteins to those of randomly sampled, non-interacting protein pairs where one protein is from the input and one is from the transmission layer (Figure 4C). Strikingly, the differences in variability of interacting protein pairs are significantly higher than for those of non-interacting protein pairs (*P*<10^−16^; Wilcoxon-Mann-Whitney). We can reproduce the same results when permuting the links between randomly sampled interacting protein pairs between the input and the transmission layer. This demonstrates that the observed differences can be attributed to both the different network positions of proteins in signaling pathways and a tendency of variable input layer proteins to physically interact with stably expressed transmission layer proteins.

These observations suggest that PPIs between the input and the transmission layer might have an impact on the tissue-specificity of signaling.

### Abundance, expression variation, and conservation of disease mutations in signaling pathways

The higher conservation of the signal transmission layer is in agreement with the bow-tie (or hourglass) model proposing the presence of a conserved core with variable input and output layers modulating the signal response (e.g., as observed in the signaling pathway downstream of EGFR [2]). The trade-off between fragility and robustness of such architecture has been discussed [5] and, hence, we studied the distribution of disease mutations with respect to protein abundance, variability and conservation.

**Figure 5:**
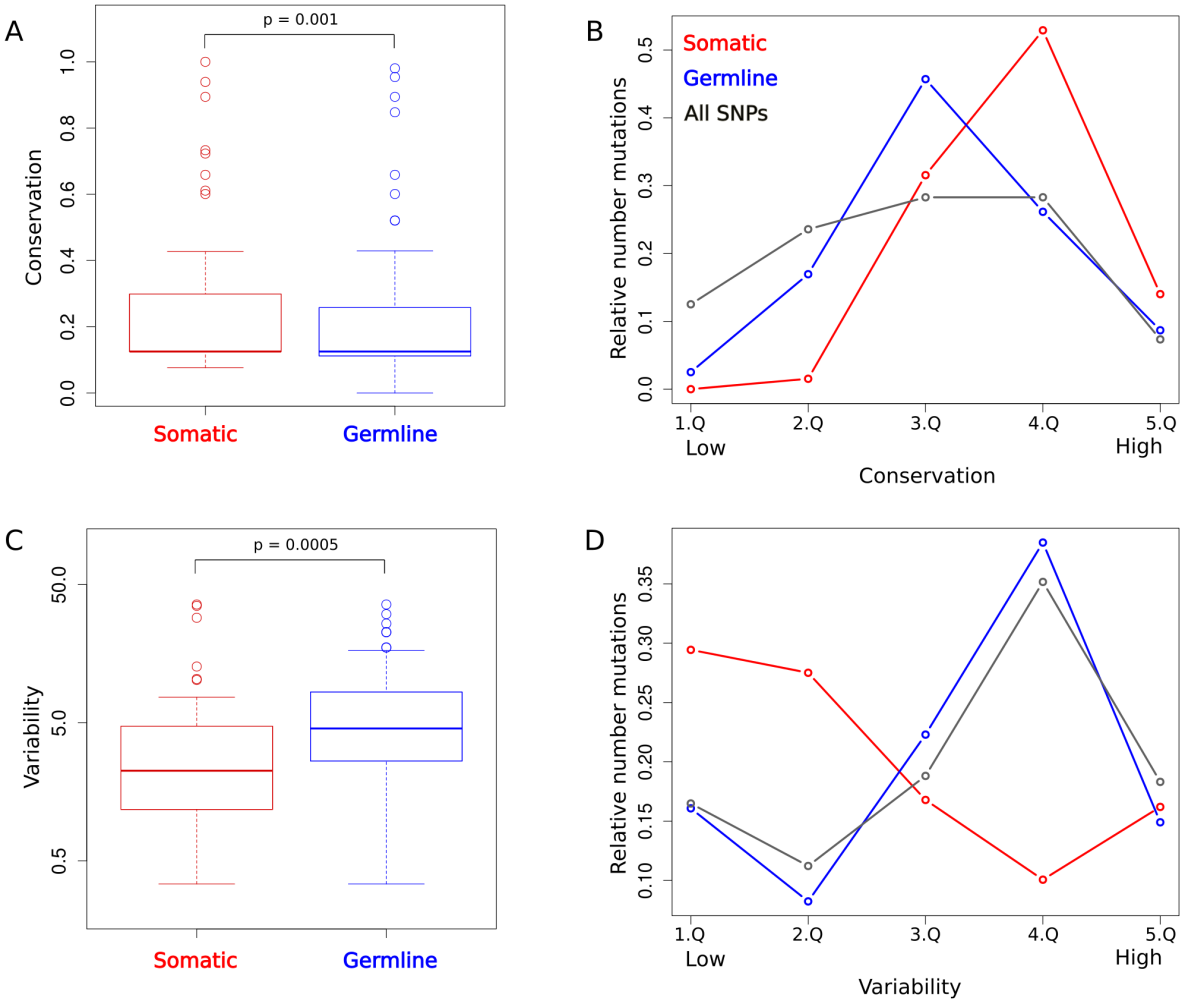
Protein conservation and variability of signaling proteins having somatic and those having germline mutations. (**A**) Protein conservation associated with signaling proteins carrying somatic disease mutations is significantly higher than for proteins with germline mutations. (**B**) The protein conservation distribution is split into five approximately equal bins (Q) and the relative number of somatic (red line) and germline (blue line) mutations associated with proteins in each bin is computed. As a comparison the respective values for all reported SNPs from UniProt are shown (gray line). (**C**) Protein variability associated with signaling proteins carrying germline disease mutations is significantly higher than for proteins with somatic mutations. (**D**) Proteins are binned according to their variability and the relative number of somatic (red line) and germline (blue line) mutations, as well as all SNPs (gray line), is shown for each respective bin.

Both germline and somatic mutations can lead to disease by perturbation of signaling pathways, e.g. in cancer [19]. Therefore, we investigated the dependency between different mutation types and protein abundance, variability and conservation. We found signaling proteins with somatic mutations to be significantly higher conserved than proteins with germline mutations (*P* = 0.001; Wilcoxon-Mann-Whitney; see Figure 5A). Additionally, we investigated how the average number of mutations changes for proteins in different conservation intervals (Figure 5B). We found that both the average number of somatic and the average number of germline mutations peak for intermediate conservation values with the distribution of somatic mutations being shifted towards higher conservation values (resulting in the observed higher conservation values associated with somatic mutations). To compare the distributions of disease mutations to background mutation rates, we also computed the average number of all reported single nucleotide polymorphisms (SNPs) in UniProt associated with the different conservation intervals. This distribution does not peak as sharply as the two disease mutation distributions and is higher for low conservation values. To investigate functional causes for the unexpected depletion of mutations for extreme values of conservation, we computed enrichment of functional categories in the sets of very lowly and very highly conserved proteins (see Methods). Among the most highly conserved proteins functions with the strongest enrichment were related to protein ubiquitination and the proteasome complex (*P* = e−^30^). The low occurrence rates for all mutation categories (somatic, germline and all SNPs) indicate that no mutations are tolerated in these proteins to maintain cellular integrity. Among the lowly conserved proteins the most strongly enriched functions were all related to sensory and olfactory perception (*P* = e^−195^). The lower rate of disease mutations as compared to all SNPs within this group likely reflects the tolerable effect of mutations within the sensory system on cell viability.

With respect to protein expression, we found that signaling-related proteins with somatic cancer mutations have a significantly lower protein abundance variability (*P* = 0.0005; Wilcoxon-Mann-Whitney; see Figure 5C) as those with germline mutations. The distributions of mean number of somatic and germline mutations associated with different intervals of variability values show opposing behavior to each other (Figure 5D): While low variability values are associated with high numbers of somatic mutations, the mean number of germline mutations peak for larger variability values (before the number of germline mutations drops for the highest variability interval).

We also found a weak though significant tendency for disease proteins with somatic mutations to be more highly expressed than proteins with germline mutations in signaling pathways (*P*<0.05; Wilcoxon-Mann-Whitney).

Taken together these observations support our previous hypothesis that the stably expressed and conserved core signaling pathway may perform a fundamental general role in development and generally in many cell types, and therefore germline mutations seem to be not tolerated. In contrast, non-core proteins, which tend to be expressed more cell type-specific, may tolerate germline mutations to a larger extend, presumably as the causing diseases will affect only some tissues.

## Discussion

We present here a systematic study of signaling proteins with respect to protein level abundances and evolutionary conservation. By doing so, we can confirm previous observations (mainly based on mRNA levels) but also provide novel hypotheses on the organization of human signaling pathways (as discussed in the following). Some of our central findings are drawn from the analysis of protein abundance variability across cancer cell lines. As we are here interested in studying normal cellular properties, we demonstrate that there is a good agreement between protein variability across cancer cell lines and across normal cells.

We report significant correlations between phylogenetic conservation and both protein abundance (positive correlation) and abundance variability (negative correlation). These observations suggest on one hand that evolutionary conserved proteins could have an essential general function for every cell type (see Figure 1C). This is in agreement with previous proteomics studies [20], [21] identifying a central proteome of ubiquitously and abundantly expressed proteins, which are correlated in their abundances across different species. This central proteome was found to have a higher than average conservation. On the other hand, recent proteins exhibit less abundance and more cell to cell type variability, suggesting they should be more involved in cell type-specific differences. This agrees with previous studies reporting that genes with RNA expression profiles restricted to a small number of mouse tissues tend to be metazoan-specific [22], [23].

It has been observed before [6] that tissue-specific proteins tend to interact with universally expressed proteins. To elucidate mechanisms by which the interactions between tissue-specific and general housekeeping proteins lead to tissue-specific modulation of signaling pathways, we investigated patterns of protein expression and conservation among signaling pathways. An important implication of our analyses is that the interactions between receptors and cytoplasmic proteins might have the strongest impact on the modulation of tissue-specificity of signaling. We observe a larger difference in protein abundance variability between signaling proteins associated with the input and the transmission layer than, for example, between cofactors and mediators within the transmission layer. This difference is even stronger for proteins that physically interact.

The decreasing protein abundance variability from the input to the output layer might be surprising (especially since in many of the known cases the cell type-specific response to signaling pathway activation depends on the abundance of transcriptional cofactors; see Introduction). However, the low variability of the output layer is additionally supported by our observation that cellular processes related to gene expression have the lowest variability among all cellular processes. Also, it is in agreement with previous studies reporting a lower mRNA variation for intracellular signaling components [24] and demonstrating how different cell types recruit a common effector network to determine the cellular response [25].

Several computational and experimental studies suggested the presence of a core signaling backbone (e.g., [25], [26]), sometimes referred to as the hourglass or bow-tie model of signaling [4], [5] to emphasize how signals converge from a larger input onto a conserved core. However, the mechanisms by which the core signaling machinery is modulated to respond in a cell type-specific way remain largely unknown. Here, we propose that an evolutionary conserved and stably expressed core of signaling pathways, which is modulated by less conserved and non-uniformly expressed receptors, extends the previous model and provides means to understand cell type-specific signaling as the consequence of a dynamic wiring logic between the input and the transmission layer.

In addition, the conserved core is re-used in different pathways as our analysis of the conservation among proteins unique to a single pathway as compared to proteins being part of multiple pathways revealed. This holds both for top-level cellular processes as well as signaling pathways.

We show how this general pathway organization principle shapes the distribution of disease mutations. As it has been discussed before [5], the bow-tie architecture confers biological systems with robustness but at the same time creates fragilities. It allows (due to the modularization and central control units for entire biological processes such as apoptosis or cell growth) its hijacking by manipulating a single or a few nodes. In PPI networks, most disease proteins are located in the network periphery and are only expressed in a limited number of tissues [27]–[29], likely due to developmental constraints selecting against mutations in central and housekeeping proteins. However, somatic mutations (not undergoing *in utero* selection) show contrary patterns and are associated to a higher degree with central and housekeeping genes [28]. In agreement, we report here that signaling proteins harboring germline mutations differ from proteins with somatic mutations with respect to protein abundance variability (and to a weaker degree in conservation and abundance). It is interesting to note that a recent study [30] found mutations in the TGF-β and Wnt/β-catenin signaling pathways to be often associated with only a single cancer type (as opposed, for example, to mutations in proteins related to genome integrity, which tend to be associated with different cancer types). This again highlights the importance of understanding the cell type-specific dynamics of signaling for the elucidation of tissue-specific disease mechanisms.

In summary, to understand cell type-specific signaling mechanisms and, more general, to understand “what makes a cell type”, we need to distinguish between core proteins conserved through evolution, and those recently acquired and incorporate information on protein concentration to interaction networks. Ideally this should be complemented by structural information to distinguish between competing and compatible interactions [31] as well as protein localization in the cell. The effect of receptor abundance on their physical interactions with members of the transmission layer (such as kinases, GTPase binding proteins and adaptors) should be a major research focus to improve the understanding of the combinatorial logic of cooperativity and competition for binding.

## Materials and Methods

### Processing of the proteomics data

We retrieved a recently published proteomics dataset [11] quantifying the abundances of almost 12,000 proteins in eleven human cell lines (A549, GAMG, HEK293, Hela, HepG2, Jurkat, K562, LnCap, MCF7, RKO, and U2OS). We standardized the given mass spectrometry intensities (by subtracting from each measurement the sample mean and dividing by the sample standard deviation) and extracted mean abundance and variance values. The mean abundance was computed averaging the standardized iBAQ values. To estimate the variability, we computed *F* values dividing the between-cell variability by the within-cell variability on the standardized label-free quantification intensities, thereby eliminating the dependence between variation and abundance (see Figure S1). Only proteins where considered that were detected in at least 50% of the MS replicates and that could be uniquely and unambiguously mapped to one protein entry in UniProt/SwissProt. For visualization purposes, distributions of *F* values are shown in logarithmic scale throughout the manuscript.

We contrasted the computed *F* values with gene expression measurements from healthy tissues. First, we retrieved a set of housekeeping genes defined based on uniformly distributed RNA abundance measurements in 16 healthy human tissues [12]. Second, we retrieved protein quantifications from 28 healthy mouse tissues [13]. As in the case of the mouse proteomics study no replicates were available, we could not normalize the inter- with the intra-sample variability. Therefore we only considered highly abundant proteins (larger than average) to minimize the confounding impact of protein abundance on variability. We also required that the proteins had been detected in all samples. We extracted the proteins falling in the lowest and the highest standard deviation quartile and mapped these proteins to their human orthologs.

### Homology prediction and gene conservation

Homology information for proteins was extracted from the NCBI database (http://www.ncbi.nlm.nih.gov/sites/entrez) using the HomoloGene search tool. We considered conservation in *Pan troglodytes*, *Mus musculus*, *Rattus norvegicus*, *Gallus gallus*, *Danio rerio*, *Drosophila melanogaster*, *Anopheles gambiae*, *Caenorhabditis elegans*, *Schizosaccharomyces pombe*, *Saccharomyces cerevisiae*, *Eremothecium gossypii*, *Arabidopsis thaliana*, and *Oryza sativa*.

A phylogenetic tree was constructed using inferred phylogenetic relationships between these species [32]. For the purpose of associating each protein with a conservation score reflecting the evolutionary distances across the species in which the protein is conserved, we associated each human protein with a pruned phylogenetic subtree containing only those species in which the protein is conserved. The conservation score was computed as the sum of all branch lengths present in the pruned subtree divided by the sum all branch lengths present in the full phylogenetic tree. In formal notation, for each protein i a pruned tree T_i_ is constructed as a subtree of the full phylogenetic tree T. Branch lengths are mapped as weights to the set of edges E. The conservation score is then computed as:
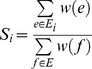
where E_i_ is the set of edges associated with subtree T_i_ and w(e) the weight corresponding to edge e. For an example of the conservation score computation see Figure S3.

### Analysis of proteins from the Reactome pathway databases

Proteins involved in the 22 top-level pathway classes in the Reactome pathways database [14] were downloaded (May 2013). Several pathways were merged or removed. The complete 22 pathways defined in Reactome are listed in Table S1 together with reasons for deletion or merging. To assign proteins from Reactome to functional classes, we retrieved functional data from GO and the UniProt Knowledgebase (UniProtKB). We considered the intersection of proteins being associated with the UniProtKB term ‘Membrane’ and those associated with the UniProtKB term ‘Receptor’ as membrane-bound receptors. Proteins indicated as being ‘DNA-binding’ in UniProtKB were considered as transcription factors. Kinase classification was also retrieved from UniProtKB. The definitions of phosphatases (GO:0016791), adaptors (GO:0035591), and GTPase binding proteins (GO:0051020) were retrieved from GO.

### Mapping of disease mutations on proteins

To construct the set of germline mutations, we retrieved all disease mutations from OMIM [33] and excluded entries labeled as somatic mutations. The set of somatic mutations was assembled by retrieving cancer mutations from COSMIC [34] including only somatic missense mutations.

### Functional enrichment among lowly and highly conserved proteins

We computed enrichment of functional categories among the proteins falling into the first and the last quantile of the conservation distribution. We used the web tool DAVID [35] to identify gene ontology terms and domains enriched among these protein groups. We used all signaling proteins as a background. Indicated enrichment P-values correspond to the Bonferroni-corrected values given by DAVID.

## Supporting Information

Figure S1 **Correction for relation between protein abundance and variability.** (A) Highly abundant proteins are associated with a lower variability (correlation = −0.62, *P*<e^−16^). (B) Correcting with intra-cell variance eliminates this dependence (after computation of the *F* value, no significant association is detected).
(TIF)

Figure S2 ***F*** **values associated with human housekeeping genes and mouse lowly and highly variable proteins.** (A) We compared the distribution of *F* values associated with housekeeping genes (defined by uniform RNA abundances over a panel of healthy human tissues) versus all other human genes. The protein products of the housekeeping genes were associated with a significantly lower *F* values than other proteins (*P*<e^−16^; Wilcoxon-Mann-Whitney). (B) We compared the distribution of *F* values associated with the least variable quarter and the highest variable quarter of mouse proteins. Protein abundances were measured in 28 healthy mouse tissues and proteins were mapped to human. The lowly variable mouse proteins are associated with lower variability across human cell lines (*P*<0.001; Wilcoxon-Mann-Whitney).
(TIF)

Figure S3
**Computation of a phylogenetic tree-based conservation score.** For each human protein we created a phylogenetic tree of species in which the protein is conserved. The conservation score is then computed as the sum of all branch lengths present in the pruned subtree divided by the sum all all branch lengths present in the full phylogenetic tree. As an example the score computation is illustrated for a protein only conserved in mammals. Blue edges constitute the phylogenetic subtree connecting mammalia (in which the protein is conserved). Branches leading to taxa in which the protein is not conserved are shown in gray. The score is computed as the sum of branch lengths associated with edges in the subtree (0.11527) divided by the sum of branch lengths in the full phylogenetic tree (2.33187). Hence, in the shown example the conservation score would be 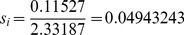.(TIF)

Figure S4 **Features of exclusive and re-used pathway proteins.** The boxplots show distributions of conservation, abundance variability, and mean abundance of proteins being part of one (Exclusive) or several (Multiple) general cellular pathways (A–C) or signaling pathways (D–F).
(TIF)

Figure S5 **Conservation, mean abundance and variability associated with different pathway classes.** The plots on the left are generated from SignaLink proteins and their annotations (Ligand – ligands; Rec – receptors; TF – transcription factors; Co-F – cofactors; Med - mediators). On the right, the respective distributions for Reactome signaling proteins and their functional annotations based on UniProt and GO are shown (Rec – receptors; TF – transcription factors; Adapt – adaptor proteins; GTP - GTPase binding proteins; Kin – kinases; Phos – phospatases).
(TIF)

Figure S6 **Protein conservation and abundance variability associated with different pathway layers of Reactome signaling proteins.** Protein conservation (A) and abundance variability (B) are shown. The input layer consists of receptors, the transmission layer of adaptors, kinases, phosphatases and GTPases and the output layer of transcription factors.
(TIF)

Table S1 **Reactome pathways.** The table lists Reactome pathways and associated protein counts (total and associated with proteomics data). The last column indicates reasons for merging pathways or excluding them from the analyses.
(XLS)
