# Supplementary figures and images for: Protein Conservation and Variation Suggest Mechanisms of Cell Type-Specific Modulation of Signaling Pathways
Source: PLoS Comput Biol. 2014 Jun 12;10(6):e1003659. doi: 10.1371/journal.pcbi.1003659 (PMC4055412; doi:10.1371/journal.pcbi.1003659)

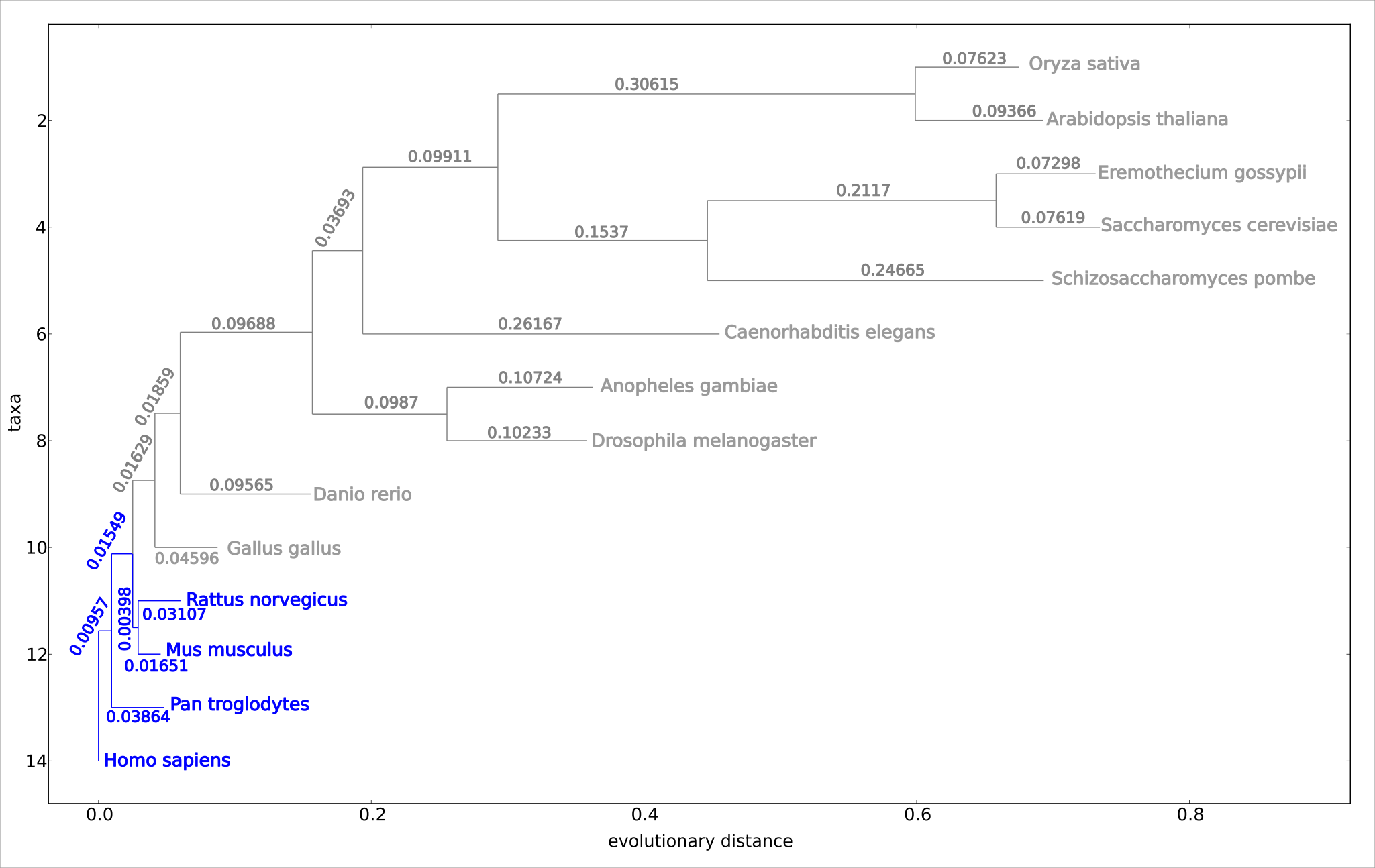

Supplement: Figure S3 — Computation of a phylogenetic tree-based conservation score. For each human protein we created a phylogenetic tree of species in which the protein is conserved. The conservation score is then computed as the sum of all branch lengths present in the pruned subtree divided by the sum all all branch lengths present in the full phylogenetic tree. As an example the score computation is illustrated for a protein only conserved in mammals. Blue edges constitute the phylogenetic subtree connecting mammalia (in which the protein is conserved). Branches leading to taxa in which the protein is not conserved are shown in gray. The score is computed as the sum of branch lengths associated with edges in the subtree (0.11527) divided by the sum of branch lengths in the full phylogenetic tree (2.33187). Hence, in the shown example the conservation score would be . (TIF) [file pcbi.1003659.s003.tif]
